# Independence and Sex Differences in Physical Activity and Sedentary Behavior Trends from Middle Adolescence to Emerging Adulthood: A Latent Class Growth Curve Analysis

**DOI:** 10.3390/ijerph19052647

**Published:** 2022-02-24

**Authors:** Yan Luo, Juan Zhong

**Affiliations:** 1 Department of Physical Education and Health Education, Springfield College, Springfield, MA 01109, USA; yluo2@springfieldcollege.edu; 2 Department of Psychology, Springfield College, Springfield, MA 01109, USA

**Keywords:** adolescent, emerging adult, health behavior

## Abstract

The purpose of this study was to examine the relationship and sex differences in the growth patterns of moderate-to-vigorous physical activity (MVPA) and sedentary behavior (SB) from middle adolescence (around age 15) to emerging adulthood (18–29 years old). We used the secondary data from the National Longitudinal Study of Adolescent Health’s (Add Health) publicly accessible database. MVPA and SB were assessed four times (1995, 1996, 2000–2001, and 2008–2009) for a total of 681 individuals from middle adolescence to emerging adulthood. Latent class growth modeling was utilized to identify heterogeneous growth patterns in MVPA and SB. Chi-square tests were used to assess group dependence and sex differences in MVPA and SB growth patterns. Seven quadratic growth patterns for MVPA and two linear growth patterns for SB were found. Both MVPA and SB growth patterns exhibited slow or rapid rate of change at different periods of adolescence and emerging adulthood. MVPA growth patterns included: decline (slow)-and-rise (rapid), rise-and-decline (both rapid), decline-and-rise (both rapid), consistently low, consistently high, decline (rapid)-and-decline (slow), and decline (rapid)-and-rise (slow). SB growth patterns included: consistently low (slow decline) and consistently high (rapid decline). While women were more likely to be involved in consistently low group for MVPA and consistently low (slow decline) group for SB, men were more likely to be involved in rise-and-decline (both rapid) group, consistently high group, and decline (rapid)-and-rise (slow) group for MVPA and consistently high (rapid decline) group for SB. SB growth patterns were independent of MVPA growth patterns. SB should not be assumed to decrease as a result of MVPA intervention. Treatment of MVPA should prioritize adolescence over emerging adulthood, with an emphasis on preventing men’s MVPA levels from decreasing in emerging adulthood and increasing women’s overall MVPA levels.

## 1. Introduction

Adolescents gain numerous physiological and psychological benefits from regular physical activity (PA) [1,2]. Sedentary behavior (SB), on the other hand, is a cause for concern during adolescence, as it is associated with many undesirable health outcomes [1,3]. Nonetheless, less than 26% of high school students in the U.S. participate in 60 min of physical activity per day [4]. Rather, roughly two-thirds of this population’s waking hours are spent participating in different types of SB [5]. This unhealthy lifestyle is likely to persist or deteriorate throughout emerging adulthood [6], which is a developmental period between 18–29 years old [7]. Emerging adulthood is a vital transitional stage marked by professional career, marriage, and parenting, all of which have been shown to have an effect on both PA [8,9] and SB [10,11]. It is critical to understand the longitudinal patterns and association between PA and SB during this time of transition in order to design effective health treatments.

The majority of research suggests that when adolescents transition to adulthood, their overall PA levels generally drop [12,13,14]. Men often have a larger decrease in PA than women [15]. Overall PA can be broken down into more specific types. One of the most studied subtypes of PA is moderate-to-vigorous PA (MVPA), which is defined as five to eight metabolic equivalents (METs; one MET equals the energy expenditure associated with quiet sitting [16]). Individual variations in MVPA change have been documented [17]. Gordon et al. found that 61% of individuals did not achieve sufficient MVPA level throughout adolescence and emerging adulthood [16]. Thirty one percent of participants had adequate MVPA in adolescence but not in emerging adulthood [16]. Only 4.4% of them maintained high engagement in MVPA in both adolescence and emerging adulthood [16]. Leisure-time PA is another subtype of PA which is often been considered a combination of light PA and MVPA. Like MVPA, leisure-time PA has also been reported to develop differently across individuals. Aaltonen et al. discovered that adolescents who were physically active throughout adolescence were more likely to suffer a decline in leisure-time PA during emerging adulthood [18]. Moderately active individuals were more prone to have an increase in leisure-time PA, while sedentary individuals maintained their leisure-time PA levels from adolescence to emerging adulthood [18]. These findings indicate that when people are divided into subgroups, PA growth from adolescence to emerging adulthood is not a homogeneous but a rather heterogeneous process across individuals (PA does not always drop or the rate of decline varies across individuals).

According to the 24-h activity cycle of daily physical behaviors [19], changes in one of the four basic activities (sleep, SB, light PA, and moderate-to-vigorous PA) will result in changes in at least one other activity. This hypothesis is supported by prior research indicating that a decrease in PA is often followed by an increase in SB [15,20,21]. However, some researchers have shown a decline in SB from adolescence to emerging adulthood [22], suggesting that SB development is also a highly heterogeneous process.

Taken together, these seemingly contradictory findings suggest that adolescents may enter adulthood via a variety of PA and SB paths. While prior research has demonstrated the diverse development processes of PA and SB on their own, little is known about their relationship. Understanding the developmental link between PA and SB is critical because it can guide intervention programs regarding whether PA change occurs concurrently with SB change. Additionally, past research viewed the development of PA and SB as a linear process (a growth process in which the direction and the rate of change remain relatively constant). Given the significant life experiences that occur between adolescence and emerging adulthood, it is likely that the growth patterns of PA and SB are non-linear (a growth process in which the direction, the rate of change, or both of them alters).

With these limitations considered, the present study aimed to examine the heterogeneous growth patterns of MVPA and SB from middle adolescence (around age 15 [23]) to emerging adulthood. Specifically, this study aimed to examine: (a) the individual differences in MVPA and SB growth patterns from middle adolescence to emerging adulthood; (b) the relationship between MVPA and SB growth patterns from middle adolescence to emerging adulthood; (c) the sex differences in MVPA and SB growth patterns from middle adolescence to emerging adulthood.

## 2. Materials and Methods

### 2.1. Study Design and Participants

The present study utilized the data from a longitudinal, population-based sample obtained from The National Longitudinal Study of Adolescent Health’s (Add Health) publicly accessible database (https://dataverse.unc.edu/dataverse/addhealth (Accessed on: 8 February 2021)). A sample of 80 U.S. high schools and 52 middle schools were chosen by the Add Health study via unequal probability of selection approach. Systematic sampling procedures and implicit stratification were used to assure representation of U.S. schools with regard to area of country, urbanicity, school size, school type, and ethnicity. The research population was comprised of more than 20,000 adolescents enrolled in grades 7 through 12, supplemented with minority special samples. Detailed study design and sampling procedure can be found elsewhere [24,25]. Multiple in-home surveys were conducted to follow participants in 1995 (wave 1), 1996 (wave 2), 2000–2001 (wave 3), and 2008–2009 (wave 4). The number of publicly accessible participants for each wave was 6504 (wave 1), 4834 (wave 2), 4882 (wave 3), and 5114 (wave 4). Individuals who participated in all four waves were selected for this study (N = 3342). The University of North Carolina at Chapel Hill’s Institutional Review Board granted ethical approval for collecting informed consent and conducting the Add Health project.

Similar to the stratification approach detailed in the Mcphie and Rawana’s study [26], we only included participants aged 15 at wave 1 in this study (N = 681) to precisely capture the developmental patterns of PA and SB through middle adolescence and emerging adulthood. Age was defined as the number of years between birth and the time of the in-home survey at wave 1 [27]. Participants mean age at wave 1 through wave 4 was 15 (SD = 0.00), 16 (SD = 0.00), 21.24 (SD = 0.43), and 28.00 (SD = 0.13) respectively.

### 2.2. Measures

#### 2.2.1. Sex

The total number of women and men included in this study were 392 (57.56%) and 289 (42.44%), respectively. We coded women as 0 and men as 1 for data analysis.

#### 2.2.2. Physical Activity

The Add Health surveys included a standard recall of MVPA habit that is comparable to previous self-report questionnaires that have been used and validated in other large-scale epidemiological trials [28,29]. Participants were asked to self-report participation in MVPA activities in the past seven days across all waves. Participants responded to questions about the frequency of 16 types of MVPA specific to adolescence at wave 1 and wave 2. At wave 3 and wave 4, participants responded to same questions from wave 1 and wave 2 questionnaires, as well as to additional questions specific to emerging adults.

At waves 1 and 2, MVPA was quantified by asking three questions about the frequency with which participants engaged in 16 different types of physical activity in the preceding seven days. These activities included rollerblading, rollerskating, skateboarding, bicycling (question 1), baseball, softball, basketball, soccer, swimming, and football (question 2), jogging, walking, karate, jumping rope, gymnastics, and dancing (question 3). Each question elicited a response from the participants in the form of one of the following options: 0 (never), 1 (once or twice), 2 (three or four times), 3 (five or more times), 6 (refused), and 8 (do not know).

At wave 3 and wave 4, MVPA was measured by asking seven questions regarding the frequency of participation in 34 types of MVPA in the past 7 days, which included: bicycling, skateboarding, dancing, hiking, hunting, yard work (question 1), rollerblading, rollerskating, downhillskiing, snowboarding, playing racquet sports, aerobics (question 2), football, soccer, basketball, lacrosse, rugby, field hockey, ice hockey (question 3), running, wrestling, swimming, cross-country skiing, cycle racing, martial arts (question 4), gymnastics, weight lifting, strength training (question 5), golf, fishing, bowling, softball, baseball (question 6), walking (question 7). Participants responded to each question by choosing one of the following options: 0 (not at all), 1 (one time), 2 (two times), 3 (three times), 4 (four times), 5 (five times), 6 (six times), 7 (seven or more times), 96 (refused), and 98 (do not know).

The total score of MVPA at wave 1 and wave 2 was calculated by adding the scores from question 1 through question 3 together. We transformed participants’ responses in MVPA questions at wave 3 and wave 4 according to the following rules: recoding 2 to 1; recoding 3 and 4 to 2; recoding 5, 6, and 7 to 3. This transformation approach resulted in identical coding schemes across four waves. We then divided the total frequency of MVPAs (sum of question 1 through 7) reported by each participant at wave 3 and 4 by the number of activities included in those questions and then multiplied by the number of activities included in questions at wave 1 and 2. The resulting scaled total scores at wave 3 and wave 4 were comparable to those at wave 1 and wave 2. Similar MVPA assessments and score transformation strategy were used in previous studies [16,30,31]. Table 1 shows the link between the MVPA total score, the response patterns, and the bouts of MVPA per week. Under the response pattern column, each number reflects the response to a given MVPA question. Three numbers form a specific response pattern. Only one of the response patterns with same numbers but different order was arbitrarily listed because the sequence of the responses has no effect on the overall MVPA score. For instance, response patterns 123, 132, and 213 each have the same total MVPA score 6 such that only response pattern 123 was arbitrarily listed in the table. The lower range of MVPA bouts per week for each response pattern was calculated by adding the smallest bouts associated with each response, and the upper range was derived by adding the maximum bouts associated with each response in a response pattern. When students declined to answer a question or did not know the answer, their answers were considered as missing values. There was a total of 24 (3.52%) and 14 (2.06%) missing values of MVPA at wave 3 and wave 4 respectively.

#### 2.2.3. Sedentary Behavior

SB was measured by three questions across four waves [32]: “How many hours a week do you watch television?” “How many hours a week do you watch videos?” and “How many hours a week do you play video or computer games?” The scores of all questions reported by each participant at each wave were summed and divided by 7. The resulting score represented the SB time (hours/day) for each participant. Participant answers were considered missing if they refused to answer or did not know the answer. There was a total of 2 (0.29%), 4 (0.59%), 11 (1.62%), 30 (4.41%) missing values of SB at wave 1, 2, 3, and 4, respectively. Previous research has used similar approach to assess SB level for the participants in the Add Health study [16,30].

### 2.3. Data Analysis

We used R [33] for data preparation and Mplus [34] for data analysis. Full information maximum likelihood estimation was used to deal with missing data. We first fitted the single-group linear and quadratic latent growth curve model (LGCM) to MVPA and SB separately. Different factor loadings that were proportionate to the time intervals between adjacent waves were used to account for the unequal spacing of time between assessments (wave 1 = 0, wave 2 = 0.1, wave 3 = 0.6, wave 4 = 1.3). Multiple goodness of fit indices were used to assess model fit: chi-square (p($\chi^{2}$) value greater than 0.05), comparative fit indices (CFI, greater than 0.90 [35]), Tucker-Lewis index (TLI, greater than 0.90 [35]), standardized root mean squared residual (SRMR, less than 0.08 [36], and root mean square error of approximation (RMSEA, less than 0.10 [37]). To test if model fit surpasses model complexity, the Akaike Information Criterion (AIC) and Bayesian Information Criterion (BIC) were utilized. There is no standard criterion for determining the adequacy of AIC and BIC values, but lower values often indicate a better trade-off between model fit and complexity [38].

Then, we used the latent class growth modeling (LCGM [39]) to identify the number of heterogeneous subgroups within the best-fit single-group model (linear or quadratic model) of MVPA and SB. We started with a two-class LCGM and incrementally raised the number of latent classes by 1 until the optimal class model was determined. To determine the fit of two nearby models, the Akaike Information Criterion (AIC), the Bayesian Information Criterion (BIC), the Lo-Mendell-Rubin likelihood ratio test (LMR-LRT), and the boot-strapped LRT (BLRT) were utilized. Lower AIC and BIC values, as well as p values less than 0.05 for LMR-LRT and BLRT, indicate better model fit [40]. In BLRT, 100 bootstraps were used, as previously suggested [41]. The LCGM model’s classification accuracy was determined using entropy. 0.40, 0.60, and 0.80 entropy values indicate low, medium, and high classification accuracy [42]. Additionally, we excluded any model with a sample size of less than 5% of the total participants for the smallest class, even if other fit indices support acceptance [40]. Lastly, we assessed the relationship between sex and the growth classes of MVPA and SB by using chi-square test ($\chi^{2}$) with *p* value less than 0.05 indicating sex and class dependence.

## 3. Results

### 3.1. Descriptive Statistics

Table 2 shows the average MVPA and SB levels stratified by sex across four waves. Welch’s t-test indicates that men have significantly higher level of MVPA and SB compared to women at each wave (*p* < 0.05).

### 3.2. Fit Indices for Latent Growth Curve Models

Table 3 shows the fit statistics for the linear and quadratic LGCM model for MVPA and SB, respectively. The linear growth model poorly fitted MVPA, but the quadratic model demonstrated an excellent fit to MVPA (p($\chi^{2}$) > 0.05, CFI = 1.00, TLI = 1.00, SRMR < 0.01, RMSEA < 0.001). The decrease of AIC and BIC from the linear to quadratic model indicates that gains in model fit outperforms the increase of model complexity after including the quadratic term to the model. The linear growth model for SB demonstrates an acceptable fit (p($\chi^{2}$) < 0.05, CFI = 0.917, TLI = 0.90, SRMR = 0.05, RMSEA = 0.089). Following the inclusion of a quadratic term, the TLI and RMSEA values changed to an unacceptable level (TLI = 0.83, RMSEA = 0.116). Based on the examination of fit indices, we decided to use the quadratic LGCM model for MVPA and the linear LGCM model for SB in further analysis.

### 3.3. Fit Indices, Classification Accuracy, and Group Size for Latent Class Growth Models

Table 4 shows the fit indices, classification accuracy, and group sizes of the LCGM model for MVPA and SB, respectively. Except for a small rise in AIC values from the single-group model to the 2-subgroup model for MVPA, the AIC and BIC values decreased as the number of subgroups rose. Despite that the LMR-LRT value no longer was significant in the five-subgroup model, we decided to increase the number of subgroups since the AIC and BIC values continued to drop and the BLRT value remained significant. We stopped at the 8-subgroup model as the numbers of people in groups 3 and 7 were less than 5% of the total. We determined that the 7-subgroup LCGM was the best model for MVPA. The 7 sub-group MVPA model’s entropy value suggests a medium-to-high classification accuracy (entropy = 0.75). As the number of sub-groups grew, the AIC and BIC values decreased for the linear SB models. We selected the 2-subgroup model as the best-fit model since group 2 had fewer than 5% of the participants in the 3-subgroup model for SB. The 2-subgroup SB model’s entropy score (entropy = 0.92) indicates a high classification accuracy.

### 3.4. Physical Activity and Sedentary Behavior Growth Patterns

**Figure 1:**
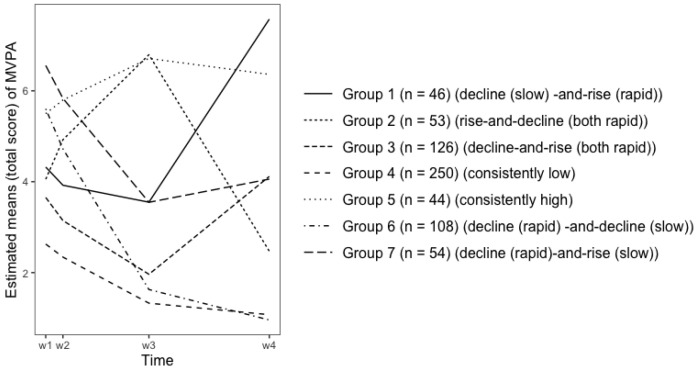
Physical activity growth patterns.

**Figure 2:**
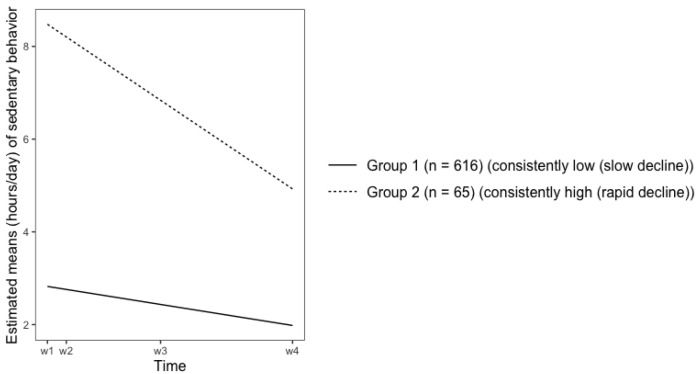
Sedentary behavior growth patterns.

Table 5 demonstrates the estimated MVPA and SB means for each group across waves. Figure 1 illustrate the 7 growth patterns for MVPA: decline (slow)-and-rise (rapid) (group 1), rise-and-decline (both rapid) (group 2), decline-and-rise (both rapid) (group 3), consistently low (group 4), consistently high (group 5), decline (rapid) -and-decline (slow) (group 6), decline (rapid)-and-rise (slow) (group 7). Figure 2 illustrates the 2 growth patterns for SB: consistently low (slow decline) (group 1) and consistently high (rapid decline) (group 2).

### 3.5. Relationship between Sex, Physical Activity Growth Patterns, and Sedentary Behavior Growth Patterns

$\chi^{2}$ tests were used to examine the relationship between the group membership of MVPA, SB, and sex. The $\chi^{2}$ test did not find significant relationship between MVPA and SB group membership ($\chi^{2}$(df) = 3.88 (6), *p* > 0.05). There was a significant sex difference in MVPA group membership ($\chi^{2}$(df) = 75.96 (6), *p* < 0.001) and SB group membership ($\chi^{2}$(df) = 6.17 (1), *p* < 0.05). Posthoc comparisons revealed that women are more likely to be involved in growth group 4 for MVPA (*p* < 0.001) and group 1 for SB (*p* = 0.05), whereas men are more likely to be involved in growth group 2 (*p* < 0.05), group 5 (*p* < 0.001), and group 7 (*p* < 0.01) for MVPA and group 2 for SB (*p* = 0.05).

## 4. Discussion

The current research investigated the individual and sex differences and relationship in the growth patterns of MVPA and SB from middle adolescence through emerging adulthood. We found that the quadratic growth pattern matched MVPA well and SB followed a linear growth pattern. There were seven and two growth patterns found for MVPA and SB, respectively. MVPA growth patterns included: decline (slow)-and-rise (rapid), rise-and-decline (both rapid), decline-and-rise (both rapid), consistently low, consistently high, decline (rapid) -and-decline (slow), and decline (rapid)-and-rise (slow). SB growth patterns included: consistently low (slow decline) and consistently high (rapid decline). While women were more likely to be involved in consistently low group for MVPA and consistently low (slow decline) group for SB, men were more likely to be involved in rise-and-decline (both rapid) group, consistently high group, and decline (rapid)-and-rise (slow) group for MVPA and consistently high (rapid decline) group for SB. MVPA growth patterns were independent of SB growth patterns.

To address the observed heterogeneity in MVPA growth among people across middle adolescence and emerging adulthood [17], the current study identified seven different MVPA growth patterns. Regardless of decline rate, 87% of individuals (sum of groups 1, 3, 4, 6, and 7, see Table 4) reported a decline in MVPA in late adolescence (wave 1 to wave 3). MVPA began to recover for 39% of them in emerging adulthood (sum of groups 1, 3, and 7 divided by sum of groups 1, 3, 4, 5, 6, and 7; wave 3 to wave 4, see Table 4), while 61% continued to experience varied declines in MVPA (sum of groups 4 and 6 divided by sum of groups 1, 3, 4, 6, and 7, see Table 4). These findings corroborate prior research indicating that MVPA usually declines as adolescents enter emerging adulthood [12,13]. Additionally, the current study noted that MVPA change is more heterogeneous in emerging adulthood than in adolescence. 67% (sum of groups 2, 4, 5, and 6, see Table 4) of participants dropped and 34% (sum of groups 1, 3, and 7, see Table 4) of them increased in MVPA during emerging adulthood. Similar daily routines and life events (e.g., schooling, socializing, and playing) may account for the low heterogeneity of MVPA change during adolescence, but the noteworthy heterogeneity of MVPA change in emerging adulthood may be explained by participants’ unique life experiences (e.g. occupation, marriage and employment status).

Despite minor variations, individuals in groups 4 and 5 (43%, see Table 4) maintained their MVPA levels throughout adolescence and emerging adulthood. While individuals in group 4 (37%, see Table 4) were persistently inactive, those in group 5 were habitual exercisers (6%, see Table 4). Women are more likely to be in group 4 and men are more likely to be in group 5. This implies that men tend to sustain a higher level of MVPA throughout middle adolescence and emerging adulthood compared to women. This conclusion is consistent with prior research [21,43,44,45,46]. Although participants in groups 1, 3, and 7 showed a decrease and a comeback in MVPA levels between middle adolescence and emerging adulthood (see Figure 1), MVPA levels remained significantly lower in group 7 at the end of emerging adulthood than at the outset (see Figure 1). Similar to group 7, adolescents in group 2 exhibited a dramatic drop in MVPA, which happened in emerging adulthood rather than in youth. The fact that men tend to fall into groups 2 and 7 suggests that men who have a high level of MVPA in middle adolescence may have a sharp decline in MVPA later on, although the timing of the decline varies across individuals. The most dramatic drop in MVPA occurred in group 6, where 16% of people (see Table 4) transitioned from a high level of MVPA in mid-adolescence to a low level in emerging adulthood. In terms of both quantity (MVPA declined for individuals in groups 3 and 4 in late adolescence, accounting for 56% of total participants, see Table 4 and Figure 1) and severity (group 6 had the steepest MVPA decline in late adolescence, accounting for 16% of total participants, see Table 4 and Figure 1), MVPA promotion programs should prioritize adolescence over emerging adulthood.

The present study found that SB decreased substantially from middle adolescence to emerging adulthood, which is inconsistent with previous findings [16]. These contradictory findings might be the results of two reasons: (1) the present study used 28 years old as the ending point of emerging adulthood, whereas the other study used 22 years old [16]; (2) the SB measure in this study only covered a limited range of SB activities, other activities common for adolescents, such as time spent in studying and web browsing were not included. More studies should be conducted to add more evidence in the overall SB change in this transitional period. In addition, we found that individuals who had a higher level of SB in middle adolescence were more likely to experience a greater drop of SB than individuals who had a lower SB level in middle adolescence (see Figure 2). Men are more likely to be involved in the “consistently high group” v.s. “consistently low group (slow decline)”, suggesting that men are more sedentary in their middle adolescence, but their sedentary behavior dropped quickly when entering emerging adulthood.

From adolescence to emerging adulthood, we discovered that MVPA growth is independent of SB growth. This result is consistent with a prior study that found that not every adolescent who watches an excessive quantity of television is inactive [47]. These results indicate that a subset of adolescents and young adults may engage in significant amounts of MVPA while also partaking in considerable amounts of SB. Additional research should be conducted to ascertain the health advantages and hazards associated with this specific way of life in order to determine whether intervention programs should be tailored to this particular lifestyle. Furthermore, these results indicate that treatments aimed at increasing MVPA level should not make the assumption that SB would decrease as a result of the intervention. SB level must still be carefully monitored to ensure that MVPA intervention has an impact on it as well.

The present study has three major strengths. To begin, LCGM is a relatively new statistical tool that has been utilized sparingly in the physical activity literature. To our knowledge, this is the first study to apply LCGM to classify the diverse growth patterns of MVPA and SB from middle adolescence to emerging adulthood. Secondly, the longitudinal association between MVPA and SB growth has remained largely unexplored. The current study established longitudinal evidence of independence between MVPA and SB growth patterns. Thirdly, the data analyzed in this study originate from a large-scale investigation in which nationally representative participants were recruited. As a result, the study’s findings have a high degree of external validity for the population studied.

The present study has few limitations. Firstly, the time frame of this study ranged from 1995 to 2009. During this period, many technological advances boomed, such as personal laptops and video games. Hence, the MVPA and SB growth trajectory identified in this study may not be representative for other time periods. Secondly, MVPA and SB in this study were measured by participants’ self-reporting. Potential measurement bias by using this method has been well documented in previous literature [47,48].

## 5. Conclusions

The present study investigated the longitudinal growth patterns, association, and sex differences in MVPA and SB from middle adolescence to emerging adulthood. Our findings have demonstrated that both growth patterns of MVPA and SB are heterogeneous. MVPA demonstrated a non-linear growth pattern (quadratic), whereas SB demonstrated a linear growth pattern. Men and women both exhibited an inclination for certain MVPA and SB growth patterns. MVPA growth patterns are independent of SB growth pattern. MVPA treatment should emphasize adolescence over emerging adulthood, with a focus on preventing men’s MVPA levels from declining in emerging adulthood and boosting women’s total MVPA levels. SB should not be assumed to diminish as a consequence of MVPA intervention.

## Figures and Tables

**Table 1:** Interpretation of the total MVPA scores.

Total Score	Response Pattern	Bouts of MVPA per Week	Interpretation
0	000	0	0 bouts/week
1	100	1–2	1–2 bouts/week
2	110 or 200	2–4 or 3–4	2–4 bouts/week
3	120 or 300 or 111	4-6 or >5 or 3–6	>3 bouts/week
4	130 or 220 or 112	> 6 or 6–8 or 5–8	>5 bouts/week
5	230 or 113 or 122	> 8 or >7 or 7–10	>7 bouts/week
6	123 or 222 or 330	> 9 or 9-012 or >10	>9 bouts/week
7	133 or 223	>11	>11 bouts/week
8	233	>13	>13 bouts/week
9	333	>15	>15 bouts/week

**Table 2:** Descriptive statistics of physical activity and sedentary behavior.

Variable	N	Overall	Women	Men	Difference	95% CI	*p*
MVPA (total score)							
Wave 1	681	4.02 (2.11)	3.50 (1.98)	4.73 (2.07)	−1.2	−1.5, −0.92	<0.001
Wave 2	681	3.75 (2.09)	3.26 (1.93)	4.43 (2.10)	−1.2	−1.5, −0.87	<0.001
Wave 3	657	2.57 (2.43)	2.03 (2.11)	3.31 (2.64)	−1.3	−1.7, −0.90	<0.001
Wave 4	667	2.73 (2.36)	2.43 (2.16)	3.14 (2.55)	−0.71	−1.1, −0.34	<0.001
SB (hours/day)							
Wave 1	679	3.43 (2.87)	3.18 (2.65)	3.77 (3.12)	−0.59	−1.0, −0.14	0.010
Wave 2	677	3.07 (3.00)	2.77 (2.61)	3.50 (3.42)	−0.73	−1.2, −0.26	0.003
Wave 3	670	3.15 (2.88)	2.90 (2.78)	3.51 (2.98)	−0.61	−1.1, −0.17	0.007
Wave 4	651	2.17 (2.44)	1.91 (2.18)	2.53 (2.73)	−0.62	−1.0, −0.23	0.002

The mean (standard deviation) of MVPA total scores and hours of SB per day are presented. See Table 1 for the interpretation of the MVPA total scores in the unit of bouts/week; CI = confidence interval.

**Table 3:** Fit indices and information criteria for latent growth curve models.

Model	$\chi^{2}$(df)	CFI	TLI	SRMR	RMSEA (90% CI)	AIC	BIC
MVPA							
Linear	80.93(5) ***	0.77	0.73	0.07	0.149 (0.122–0.179)	11,702	11,742
Quadratic	0.79(1)	1	1.004	0.01	0 (0–0.097)	11,629	11,688
SB							
Linear	31.76(5) ***	0.92	0.90	0.05	0.089 (0.061–0.119)	12,816	12,857
Quadratic	10.11(1) **	0.97	0.83	0.03	0.116 (0.059–0.185)	12,802	12,861

*** *p* < 0.001; ** *p* < 0.01; CI = confidence interval.

**Table 4:** Fit indices of the best-fit latent growth curve model and latent class growth models.

Model	AIC	BIC	LMR-LRT	BLRT	Entropy	Group Size							
						Group 1	Group 2	Group 3	Group 4	Group 5	Group 6	Group 7	Group 8
MVPA													
1 group	11,629	11,688	-	-	-	-	-	-	-	-	-	-	-
2 subgroups	11,634	11,683	333.31 **	333.31 ***	0.77	496 (73%)	185 (27%)	-	-	-	-	-	-
3 subgroups	11,542	11,610	99.53 ***	99.53 ***	0.69	170 (25%)	143 (21%)	368 (54%)	-	-	-	-	-
4 subgroups	11,493	11,579	57.15 *	57.15 ***	0.71	101(15%)	88 (13%)	139 (20%)	353 (52%)	-	-	-	-
5 subgroups	11,458	11,562	42.82	42.82 ***	0.72	65 (10%)	56 (8%)	330 (48%)	91 (13%)	139 (20%)	-	-	-
6 subgroups	11,427	11,550	38.82	38.83 ***	0.74	115 (17%)	47 (7%)	121 (18%)	71 (10%)	268 (39%)	59 (9%)	-	-
7 subgroups	11,397	11,537	38.68	38.68 ***	0.75	46 (7%)	53 (8%)	126 (19%)	250 (37%)	44 (6%)	108 (16%)	54 (8%)	-
8 subgroups	11,382	11,540	23.00	23.00 ***	0.77	131 (19%)	52 (8%)	27 (4%)	238 (35%)	112 (16%)	42 (6%)	28 (4%)	51 (7%)
SB													
1 group	12,816	12,857	-	-	-	-	-	-	-	-	-	-	-
2 subgroups	12,725	12,767	399.02	399.02 ***	0.92	616 (90%)	65 (10%)	-	-	-	-	-	-
3 subgroups	12,440	12,494.12	291.11 *	291.11 ***	0.96	51 (7%)	10 (1%)	620 (91%)	-	-	-	-	-

*** *p* < 0.001; ** *p* < 0.01; * *p* < 0.05.

**Table 5:** Estimated means for physical activity and sedentary behavior.

Latent Class	Wave 1	Wave 2	Wave 3	Wave 4
MVPA (total score)				
Group 1	4.32	3.92	3.55	7.58
Group 2	4.05	4.92	6.79	2.47
Group 3	3.65	3.15	1.96	4.12
Group 4	2.63	2.34	1.33	1.07
Group 5	5.50	5.80	6.71	6.36
Group 6	5.60	4.72	1.63	0.96
Group 7	6.56	5.83	3.55	4.06
SB (hours/day)				
Group 1	2.82	2.76	2.43	1.98
Group 2	8.48	8.20	6.84	4.93

See Table 1 for the interpretation of the MVPA total scores in the unit of bouts/week.

## Data Availability

The data can be accessed via the Add Health official website https://addhealth.cpc.unc.edu/data/#public-use Accessed on: 8 February 2021.
